# A question of scent: lavender aroma promotes interpersonal trust

**DOI:** 10.3389/fpsyg.2014.01486

**Published:** 2015-01-13

**Authors:** Roberta Sellaro, Wilco W. van Dijk, Claudia Rossi Paccani, Bernhard Hommel, Lorenza S. Colzato

**Affiliations:** Cognitive Psychology Unit and Leiden Institute for Brain and Cognition, Leiden UniversityLeiden, Netherlands

**Keywords:** interpersonal trust, cognitive-control state, aromas, lavender, peppermint

## Abstract

A previous study has shown that the degree of trust into others might be biased by inducing either a more “inclusive” or a more “exclusive” cognitive-control mode. Here, we investigated whether the degree of interpersonal trust can be biased by environmental factors, such as odors, that are likely to impact cognitive-control states. Arousing olfactory fragrances (e.g., peppermint) are supposed to induce a more exclusive, and calming olfactory fragrances (e.g., lavender) a more inclusive state. Participants performed the Trust Game, which provides an index of interpersonal trust by assessing the money units one participant (the trustor) transfers to another participant (the trustee), while being exposed to either peppermint or lavender aroma. All participants played the role of trustor. As expected, participants transferred significantly more money to the alleged trustee in the lavender as compared to the peppermint and control (no aroma) conditions. This observation might have various serious implications for a broad range of situations in which interpersonal trust is an essential element, such as cooperation (e.g., mixed-motives situations), bargaining and negotiation, consumer behavior, and group performance.

## INTRODUCTION

Interpersonal trust is one of the most important determinants of initiating, forming, and maintaining social relationships (Balliet and Van Lange, 2013). As it facilitates important social behaviors, such as social bonding and cooperative behavior, it is often regarded as the social glue of society (Pruit and Kimmel, 1977; Yamagishi, 1986; Grovier, 1997; Van Lange et al., 2011). Therefore, increasing our knowledge about the factors that influence interpersonal trust is crucial for a better understanding of social life.

Earlier studies indicate that interpersonal trust is a rather volatile state that is sensitive to and adjusts to the situation at hand. Research has shown that the degree to which people trust each other is influenced, for example, by their mood (Capra, 2004) or self-construal (Maddux and Brewer, 2005). More recent studies have demonstrated that interpersonal trust increases by administering the food supplement L-Tryptophan, the biochemical precursor of serotonin (Colzato et al., 2013), and the neuropeptide oxytocin (Kosfeld et al., 2005). Moreover, Tops et al. (2013) reported trust scores to increase with salivary oxytocin levels under conditions of social novelty, but to decrease with such levels under conditions of social familiarity.

In a seminal study, Baron (1997) showed that prosocial behavior (i.e., by retrieving a dropped pen or providing change for money) was significantly greater in the presence of pleasant fragrances than in their absence.

In the present research, we examined whether interpersonal trust can be influenced by specific odors in the environment that are likely to impact cognitive-control states. Research has suggested that calming scents, like lavender (Diego et al., 1998; Field et al., 2005; Lehrner et al., 2005), bias individuals’ attention toward inclusive representational levels, whereas stimulating scents, such as peppermint (Kovar et al., 1987; Warm and Dember, 1990), bias it to exclusive ones (see Herz, 2009; Johnson, 2011, for reviews). For instance, peppermint aroma has been found to improve memory (Moss et al., 2008), sustained visual attention (Warm et al., 1991), alertness in a driving simulator task (Raudenbush et al., 2009), and athletic task performance (Raudenbush et al., 2001). In contrast, lavender aroma has been shown to attenuate fatigue (Sakamoto et al., 2005), to promote behavior commitment (Grimes, 1999), and to increase the amount of time customers spend in a restaurant and the amount of purchasing (Guéguen and Petr, 2006).

Recent studies have shown that inducing particular (non-social) cognitive-control states or control styles by means of creativity tasks affects the processing of social information in a systematic ways (Colzato et al., 2013; Sellaro et al., 2014). As shown elsewhere, tasks tapping into divergent thinking are accompanied with a more “inclusive/integrative” thinking style, whereas convergent thinking has been found to be linked with a sort of “exclusive” thinking (Fischer and Hommel, 2012; Hommel, 2012). By exploiting this property, Colzato et al. (2013) showed that people are more likely to relate their own actions to that of a co-actor in the context of a divergent-thinking task than in the context of a convergent-thinking task. This implies that divergent thinking involves a cognitive-control state that promotes self-other integration. Interestingly for our purposes, Sellaro et al. (2014) showed that adopting such thinking styles affects interpersonal trust as well: interpersonal trust is more pronounced after engaging in divergent thinking as compared to convergent thinking. Considering that interpersonal trust can be enhanced by inducing a more inclusive cognitive-control state (Sellaro et al., 2014), this suggests that being exposed to the (calming) scent of lavender will result in higher interpersonal trust, while being exposed to the (stimulating) scent of peppermint will reduce it.

We tested the link between aromas and interpersonal trust by exposing healthy young adults to the scent of either lavender (i.e., relaxing aroma) or peppermint (i.e., stimulating aroma), while engaging in a social interaction (a behavioral Trust Game). As a control condition, a third group of participants was required to perform the Trust Game in a non-scented room (cf. e.g., Guéguen and Petr, 2006; Moss et al., 2008).

**FIGURE 1:**
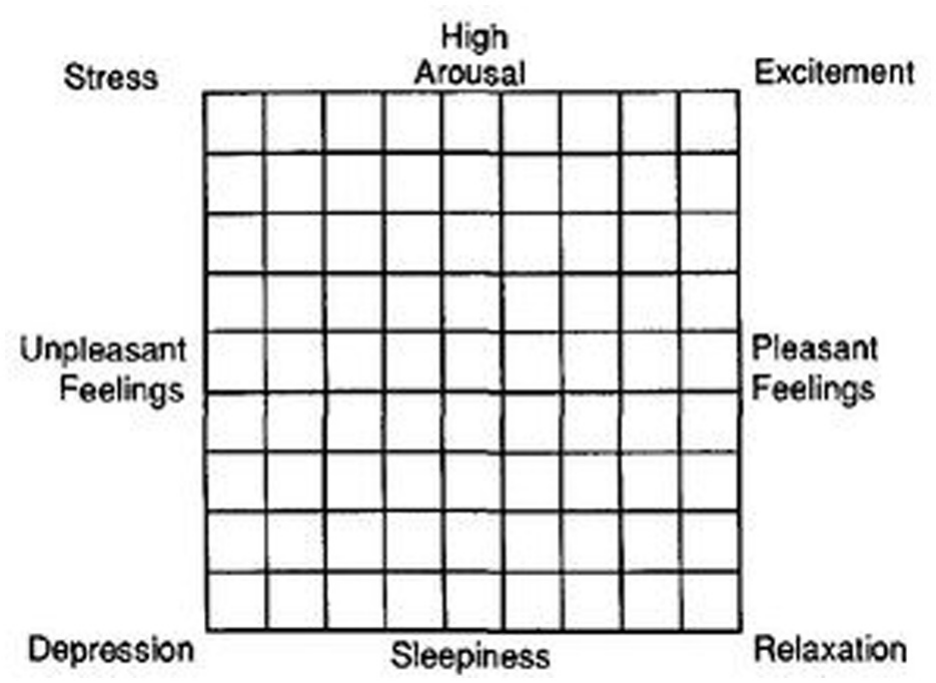
**The Affect Grid (taken from Russell et al., 1989).** The scale requires participants to rate their current affective state by placing an “X” in one of the 81 cells of the matrix. The horizontal axis represents variations in unpleasant–pleasant feelings (ranging from -4 to +4), whereas the vertical axis represents variations in arousal–sleepiness feelings (ranging from +4 to -4). The scale provides two scores, one for pleasure and one for arousal, which indicate the location of the participant’s affective state within a two-dimensional space defined by hedonic tone and activation.

Given that interpersonal trust has been found to be enhanced by positive mood (Capra, 2004) and that the exposure to pleasant aromas is reckoned to increase mood (Herz, 2009), we also assessed participants’ subjective affective states, and we did so before and after the Trust Game. To this end, we used the Affect Grid (Russell et al., 1989), a single-item scale that is particularly suitable for rapid and repeated assessment of people’s subjective affective states. The scale consists of a 9 × 9 grid, where the horizontal axis represents affective valence (ranging from unpleasantness to pleasantness), and the vertical axis represents perceived activation (ranging from high arousal to sleepiness; see **Figure 1**). Thus, two scores can be derived from the scale, one for pleasure and one for arousal. The Affect Grid has been shown to have good reliability, convergent validity, and discriminant validity (Russell et al., 1989).

## MATERIALS AND METHODS

### PARTICIPANTS

Ninety healthy young adults (mean age = 20.20 years, SD = 1.80, range = 18–24 years; 68 females) came to the lab as unacquainted same-sex dyads. Participants were screened via a phone call by the experimenter before inclusion, using the Mini International Neuropsychiatric Interview (M.I.N.I.; Sheehan et al., 1998). The M.I.N.I. is a short, structured, interview of about 15 min that screens for several psychiatric disorders and drug use, often used in clinical and pharmacological research (Sheehan et al., 1998; Colzato and Hommel, 2008; Colzato et al., 2009).

Participants were equally and randomly distributed over three experimental groups. Thirty participants played the Trust Game in a lavender-scented room, 30 in a peppermint-scented room, and the remaining participants performed the task in a non-scented room.

Prior to the testing session, participants received a verbal and written explanation of the procedure, and they were told to take part in a study investigating decision-making processes. No information was provided about the presence of aromas. At the end of the testing session participants were debriefed. Only four participants (two in the peppermint group and two in the lavender group) asked the experimenter spontaneously whether there were any aromas in the testing room but were naïve about the hypotheses concerning the outcome of the experiment.

Written informed consent was obtained from all subjects; the protocol was approved by the local ethical committee (Leiden University, Institute for Psychological Research).

### PROCEDURE

The three experimental groups [lavender, peppermint, and control (no aroma)] were tested in three different cubicles identical in size. “De Tuinen^TM^” pure essential oils (De Tuinen Aromatherapie) of peppermint and lavender were used to generate the ambient aromas. The smell of the non-scented room was odor-neutral. Following Colzato et al. (2014), four drops of the appropriate oil were applied to a candle diffuser, diluted in 30 ml of water. Two separate diffusers were used for spreading the two aromas. The diffuser was out of participants’ sight and the candle was lighted 20 min before the testing session started to assure a uniform diffusion in the testing room.

**FIGURE 2:**
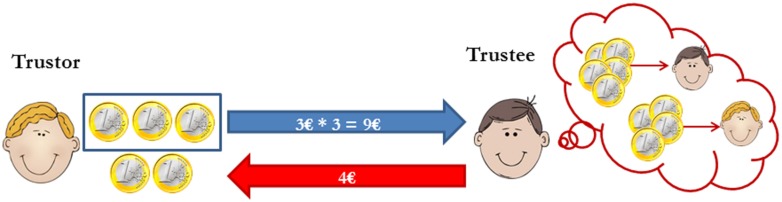
**Schematic representation of the Trust Game.** In the trust game the trustor is endowed with a certain amount of money (e.g., € 5), which s/he can keep or (partially) transfer to the trustee. Participants are told that the transferred money would be tripled and that the trustee then must decide if and how to share the new amount. The amount of money the trustor transfers to the trustee is an indicator of interpersonal trust.

Participants came to the lab as unacquainted same-sex dyads. Upon arrival, members of each dyad were seated in separate cubicles where, after having read and signed the informed consent, they were asked to rate their affective state on a 9 × 9 Pleasure × Arousal grid (values ranging from -4 to 4; i.e., The Affect Grid; Russell et al., 1989). Once they filled out the Affect Grid (i.e., after 5 min of exposure to the specific aroma), they played a behavioral Trust Game (Camerer and Weigelt, 1988; see **Figure 2**) and, immediately after, they rated again their affective state. The trust game lasted about 3 min (including instructions).

### TRUST GAME

Participants were led to believe that one of them would play the role of trustor and the other the role of trustee (in reality, all participants were trustors). Participants were endowed with 20AC 5, which they could keep or (partially) transfer to the trustee (allegedly the other member of their dyad). Participants were told that the transferred money would be tripled and that the trustee then must decide if and how to share the new amount. In this game, the amount transferred by the trustor is an indicator of interpersonal trust (Camerer, 2003).

## RESULTS

### TRUST GAME

The dependent measure was the trust score, computed as the amount of money transferred to the trustee, for each experimental group (Lavender, Peppermint, Control). To assess the effect of aroma, trust scores were submitted to a one-way analysis of variance (ANOVA) with condition (Lavender, Peppermint, Control) as between-subjects factor. As expected, aroma modulated participants’ performance in the Trust Game, *F*(2,87) = 3.53, *p* = 0.03, $\eta_{p}^{2}$ = 0.08. Fisher LSD *post hoc* tests showed that participants in the lavender-scented room transferred more money to the trustee (*M* = 3.90, SD = 125.5) than participants in both the peppermint-scented room (*M* = 3.23, SD = 115.0) [95% CI = (7.48, 125.85), *p* = 0.03], and the non-scented room (*M* = 3.20, SD = 104.5) [95% CI = (11.08, 129.45), *p* = 0.02], whose performance was comparable [95% CI = (-55.59, 62.79), *p* = 0.90].

### AFFECT GRID

Pleasure and Arousal scores were analyzed separately by means of two repeated-measures ANOVAs with effect of time (first vs. second measurement) as a within-participants factor and group (Lavender, Peppermint, Control) as a between-participants factor.

The ANOVA performed on the Pleasure scale did not reveal any significant effect or interaction between time and group, *F*s ≤ 1.90, *p*s ≥ 0.15. Pleasure levels were thus comparable across group and time: the mean scores at the two time points were 1.20 (SD = 1.5) and 1.40 (SD = 1.6) for participants in the Lavender group, 1.30 (SD = 1.1) and 1.20 (SD = 1.4) for participants in the Peppermint group, and 1.70 (SD = 1.0) and 1.80 (SD = 0.9) for participants in the Control group.

The ANOVA performed on the Arousal scale revealed a significant main effect of group, *F*(2,87) = 7.04, *p* = 0.005, $\eta_{p}^{2}$ = 0.14. Fisher LSD *post hoc* analyses showed that arousal scores were lower in the Lavender group (*M* = -0.07, SD = 1.3) than in both the Peppermint (*M* = 0.90, SD = 1.3) [95% CI = (-1.62, -0.24), *p* = 0.008] and the Control (*M* = 1.20, SD = 1.3) [95% CI = (-1.94, -0.56), *p* < 0.001] groups. Arousal levels for the Peppermint and Control groups were comparable [95% CI = (-1.01, 0.37), *p* = 0.36]. Neither a main effect of time nor an interaction between group and time was significant, *F*s ≤ 1.50, *p*s ≥ 0.25, reflecting stable arousal levels across time in all groups: the mean scores at the two time points were -0.10 (SD = 1.5) and 0.0 (SD = 1.7) for participants in the Lavender group, 0.77 (SD = 1.5) and 0.97 (SD = 2.0) for participants in the Peppermint group, and 1.07 (SD = 1.1) and 1.30 (SD = 1.3) for participants in the Control group.

To rule out the possible influence of arousal and pleasure levels in mediating the observed relationship between the degree of interpersonal trust and scent, Pearson correlations coefficients were computed between the amount of money transferred and the levels of arousal and pleasure at the first and second measurements, separately for the three groups. No significant correlations were found, *p*s ≥ 0.18, suggesting that levels of (conscious) arousal or pleasure did not affect participants’ money transfers.

## DISCUSSION

This study is the first to demonstrate that scent can have an impact on interpersonal trust. Indeed, we observed that, compared to peppermint and control (no aroma) exposure, being exposed to lavender aroma increased interpersonal trust, as indexed by the Trust Game. We argue that the calming scent of lavender temporarily induces a more inclusive cognitive-control state that, in turn, influences the extent to which people trust others. By comparison, being exposed to peppermint aroma did not reduce interpersonal trust compared to the control (no aroma) condition. This might be due to the ineffectiveness of the selected aroma to induce a more exclusive cognitive-control state to affect interpersonal trust accordingly. Alternatively, it is also possible that interpersonal trust is affected selectively by a more inclusive cognitive-control state, but not by a more exclusive cognitive-control state. Future studies might consider the idea to test whether interpersonal trust can be influenced by other aromas that, similar to peppermint, are suspected to bias cognitive-control toward a more exclusive state.

It is interesting to note that we did not find any evidence that pleasure or arousal changes might be directly responsible for the observed outcome. However, our measures relied on conscious self-assessment and thus reflect merely conscious aspects of the participant’s affective state. This does not allow us to exclude the possible impact of more implicit pleasure and arousal changes that future studies might consider by including physiological measurements, such as galvanic skin response, heart rate, and diastolic and systolic blood pressure.

The present study has some limitations that deserve discussion. First of all, we did not assess participants’ olfactory sensitivity, which would have allowed us to exclude anosmic participants and to control for potential differences in participants’ smell threshold. Thus, it is crucial for future studies to asses participants’ olfactory threshold, for example, by means of dilution-to-threshold techniques in which an odor sample is diluted with odorless air at a number of levels, and the dilution series is presented in ascending order of odor concentration. Second, we did not address explicitly whether participants were aware of the presence of the aroma, whether they could identify the specific aroma they were exposed to, and/or whether they found the scent really calming (vs. arousing). Future studies should take into consideration these important aspects. Third, it would be useful to include more objective measures to verify whether the two selected aromas differentially affected participants’ cognitive-control state. However, given that a previous study has shown that interpersonal trust may be increased by inducing a more inclusive cognitive-control state (Sellaro et al., 2014), we have reasons to believe that at least lavender aroma worked as expected. Finally, in order to control for expectancy effects, besides blinding the participants to the type of odor exposure, follow-up studies should consider to blind the experimenter in this regard as well. Moreover, given that in the current study participants met the other member of the dyad, i.e., the participant with whom they supposedly played the Trust Game, it is important for future studies to obtain evaluations regarding trustworthiness and likeability of the other member.

To sum up, these findings provide converging support for the idea that interpersonal trust is a volatile state that is, at least to some extent, controlled by domain-general (i.e., not socially dedicated) cognitive states. Moreover, the present findings reinforce the idea that interpersonal trust is sensitive to situational and environmental factors (Buchan et al., 2002; Capra, 2004; Maddux and Brewer, 2005; Colzato et al., 2013). Our results might have various serious implications for a broad range of situations in which interpersonal trust is an essential element, such as cooperation (e.g., mixed-motives situations), bargaining and negotiation, consumer behavior, social bonding, and group performance. As in the case of a previous study (Baron, 1997), which showed that prosocial behavior was significantly greater in the presence of “sweet” fragrances (e.g., baking cookies, roasting coffee), smelling the aroma of lavender may help a seller to establish more easily a trusting negotiation to sell a car, or in a grocery store it may induce consumers to spend more money buying products. The smell of lavender may also be helpful in sport psychology to enhance trust and build team spirit, for example in the case of team games such as soccer and volleyball.

## Conflict of Interest Statement

The authors declare that the research was conducted in the absence of any commercial or financial relationships that could be construed as a potential conflict of interest.
