# Corrigendum: A question of scent: lavender aroma promotes interpersonal trust

**DOI:** 10.3389/fpsyg.2015.00243

**Published:** 2015-03-10

**Authors:** Roberta Sellaro, Wilco W. van Dijk, Claudia Rossi Paccani, Bernhard Hommel, Lorenza S. Colzato

**Affiliations:** Cognitive Psychology Unit and Leiden Institute for Brain and Cognition, Leiden UniversityLeiden, Netherlands

**Keywords:** interpersonal trust, cognitive-control state, aromas, lavender, peppermint

Mistakenly, the trust scores were reported in Euros, whereas the SD and CI were reported in Eurocents.

Please find below a list of corrections made to this article:

– On page 2, right column, under RESULTS—TRUST GAME, the first sentence should read “The dependent measure was the trust score, computed as the amount of money transferred to the trustee (in Eurocents), for each experimental group (Lavender, Peppermint, Control).”
– On page 3, left column, 7th row, the text “*M* = 3.90” should read “*M* = 390.”
– On page 3, left column, 8th row, the text “*M* = 3.23” should read “*M* = 323.”
– On page 3, left column, 9th row, the text “*M* = 3.20” should read “*M* = 320.”

## Conflict of interest statement

The authors declare that the research was conducted in the absence of any commercial or financial relationships that could be construed as a potential conflict of interest.

